# Nutritional quality of new food products released into the Australian retail food market in 2015 – is the food industry part of the solution?

**DOI:** 10.1186/s12889-018-5127-0

**Published:** 2018-02-07

**Authors:** Sheree A. Spiteri, Dana Lee Olstad, Julie L. Woods

**Affiliations:** Institute of Physical Activity and Nutrition, School of Exercise and Nutrition Sciences, Deakin University, 221 Burwood Highway, Burwood, VIC 3125 Australia

**Keywords:** Food industry, Public health, Healthy eating, Food reformulation, Voluntary commitment, Food classification, New food products

## Abstract

**Background:**

Food manufacturers have made public statements and voluntary commitments, such as the Healthier Australia Commitment (HAC), to improve the nutritional quality of foods. However, limited information about the nutritional quality or healthfulness of new products makes it difficult to determine if manufacturers are doing this. The purpose of this study was to assess the healthfulness of new food products released into the Australian retail market in 2015, and whether those companies who were HAC members released healthier food options compared to non-HAC members.

**Methods:**

This cross-sectional study assessed the healthfulness of all new retail food products launched in Australia in 2015 as indexed in Mintel’s Global New Products Database. Healthfulness was assessed using three classification schemes: Healthy Choices Framework Victoria, Australian Dietary Guidelines and NOVA Food Classification System. Descriptive statistics and chi-squared tests described and compared the number and proportions of new foods falling within each of the food classification schemes’ categories for companies that were and were not HAC members.

**Results:**

In 2015, 4143 new food products were launched into the Australian market. The majority of new products were classified in each schemes’ least healthy category (i.e. red, discretionary and ultra-processed). Fruits and vegetables represented just 3% of new products. HAC members launched a significantly greater proportion of foods classified as red (59% vs 51% for members and non-members, respectively) discretionary (79% vs 61%), and ultra-processed (94% vs 81%), and significantly fewer were classified as green (8% vs 15%), core foods (18% vs 36%) and minimally processed (0% vs 6%) (all *p* < 0.001).

**Conclusions:**

This study found that the majority of new products released into the Australian retail food market in 2015 were classified in each of three schemes’ least healthy categories. A greater proportion of new products launched by companies that publicly committed to improve the nutritional quality of their products were unhealthy, and a lower proportion were healthy, compared with new products launched by companies that did not so commit. Greater monitoring of industry progress in improving the healthfulness of the food supply may be warranted, with public accountability if the necessary changes are not seen.

## Background

The prevalence of overweight and obesity continues to grow around the world at epidemic proportions [1–3]. This is concerning, as obesity has been associated with an increased risk of chronic disease [1, 4]. Population-level increases in body weight are related to a number of factors, including a decrease in physical activity, an increase in sedentary behaviours, and a shift towards greater consumption of energy-dense and nutrient-poor foods, [4, 5]. Evidence suggests that an unhealthy food environment may be a primary driver of this change in eating [6–8].

The food industry plays a central role in shaping the quality of the food environment through the production, packaging, distribution, retailing and marketing of food products [9–11]. As an integral part of the global economy, the food industry continually innovates to remain competitive and profitable [12]. The imperative to maximise profit creates conflict between the food industry and public health goals [10], as the most profitable products are typically highly processed foods that are energy-dense and nutrient-poor [10, 13–16] and are associated with increased risk of obesity and chronic disease [17]. According to the Australian Bureau of Statistics, as much as 35% of the total daily energy consumed by Australians is provided by these types of foods [18].

Governments and public health bodies have called upon the food industry to create products that are better for health [3, 19]. This has led some food manufacturers to publicly, and voluntarily, commit to improve the nutritional quality of their products and promote healthier food options through product innovation and reformulation [20–22]. In Australia, the Australian Food and Grocery Council (AFGC) has stated that ‘the entire food and grocery manufacturing industry are committed to being part of the solution’ [23]. Accordingly, the AFGC launched the voluntary Healthier Australia Commitment (HAC), in which some members pledged to improve the nutritional quality of their product portfolios by reducing sodium by 25%, saturated fat by 25% and energy by 12.5% [24]. The HAC was launched in October 2012 with the goals to be reached by 2015 and included the following AFGC members: Campbell Arnott’s, Coca Cola, General Mills, Lion, Nestle, PepsiCo, Sugar Australia and Unilever [24].

The rise in voluntary, self-regulated commitments by industry to improve their food products has created a need for independent monitoring [11, 25]. Food classification schemes that classify the nutritional quality/healthfulness of foods can play an important role in this endeavour [11, 26, 27]. These can be distinguished according to whether they use nutrient-, food- or processing-based criteria, singularly or in combination which reflect the major concepts underlying current definitions of what constitutes a “healthy” food. Many different classification schemes exist that reflect these concepts. The Healthy Choices Framework Victoria is an Australian nutrient profiling scheme that classifies foods based on the level of ‘negative’ and/or ‘positive’ nutrients into one of three categories: red foods (least healthy), amber foods (eat in moderation) and green foods (most healthy) [28, 29]. Food-based schemes such as the Australian Dietary Guidelines (ADG) classify foods in terms of their contribution to healthy dietary patterns into core (i.e. essential for health) and discretionary (i.e. not essential for health) foods [30]. The NOVA Food Classification System is a further scheme that classifies foods in accordance with their level of physical, biological and chemical processing into: minimally processed, culinary processed, processed and ultra-processed [31].

There is a dearth of information about the healthfulness of the Australian food supply and in particular, about the new products being released into it [11, 32]. This makes it difficult to determine if the food industry is producing healthier products that are consistent with their desire to be part of the solution to unhealthy eating. The purpose of this study was to assess the healthfulness of new food products released into the Australian retail food market in 2015, and to determine whether those companies who made a commitment to improve the nutritional quality of foods and promote healthier options (i.e. HAC members) released healthier food options compared to those who did not (i.e. non-HAC members). This study did not evaluate the success or otherwise of the HAC commitment, rather it uses the HAC commitment to group companies on the basis of whether they had publicly indicated an intention to produce healthier food products.

## Methods

This was a retrospective, cross-sectional study that analysed available data on all new food products (food products refers to foods and beverages – hereafter just called food(s)) launched in Australia between January 1, 2015 and December 31, 2015 that were indexed in Mintel’s Global New Products Database (GNPD). This timeframe corresponded with the final year of the HAC. Mintel’s GNPD contains a comprehensive listing of new products, including new foods, launched into the market each year [33]. The data are collected according to standardised and rigorous protocols involving a global network of expert shoppers across 62 countries [33]. New products are added daily, and more than 80 fields of information are captured for each new product [33].

The following information was extracted from the GNPD for each new food product introduced between January 1 and December 31, 2015: product description, brand, company, ingredients, energy (KJ), protein (g), fat (g), saturated fat (g), carbohydrates (g), sugar (g), fibre (g) and sodium (mg). Nutrient information was collected as per serve and as per 100 g. Given that fibre is not required to be listed on the nutrition information panel in Australia, missing values for fibre were obtained from the AUSNUT 2011–13 food composition database [34], company websites or Calorie King [35], for that particular product or for a product that was the most similar. Nutrient values were only used for one classification scheme. Only 2.8% of all foods (*n* = 117) had the fibre content missing, where it was required for classification. Seventy nine percent of these foods were biscuits and the fibre content was necessary to distinguish between a classification of amber or red. A similar process was used where there were missing data on other nutrients, e.g. saturated fat. Only 4% of all food products had some missing data (fibre, saturated fat, energy or sodium) where it was required for classification.

Foods in the GNPD are classified into the following 17 food categories: baby food, bakery, breakfast cereals, chocolate confectionary, dairy, desserts and ice-creams, fruits and vegetables, meals and meal centres, processed fish, meat and eggs, sauces and seasonings, savoury spreads, side dishes, snacks, soups, sweets and gum, sweet spreads, and sweeteners and sugar. These categories were maintained for the current analysis. Baby foods were included because on further examination they were found not to be infant foods but foods for toddlers and young children and hence were not bound by food regulation to have limited added salt or sugar. Brand and company information within the GNPD were used to determine whether the product had been launched by a HAC-member or not.

Finally, the healthfulness of new food products was assessed using three classification schemes (Table 1): 1) The Healthy Choices Framework Victoria nutrient-based criteria (green, amber, red traffic lights) [36], 2) The Australian Dietary Guidelines food-based criteria (core, discretionary, or other for products not edible on their own such as baking ingredients, herbs and spices) [17, 37], and 3) The NOVA Food Classification System level of processing criteria (minimally processed, culinary ingredients, processed, ultra-processed) [31]. This was achieved by assessing each individual food item retrieved against the criteria of each scheme. For instance, to classify according to the Healthy Choices Framework we followed the guidance it provided and classified foods by comparing each food with all of the food items listed in the Green, Amber and Red categories outlined in the framework document. Where it wasn’t clear that a food fitted into a specific category, the nutrient criteria set out in the Framework document were used to identify the correct classification. Some foods were not able to be classified within a particular scheme, for example oils are neither core foods nor discretionary foods and products such as baking powder, cream of tartar and dried herbs and spices are not considered ‘foods’ in their own right and could not be classified within the ADG or the Healthy Choices Framework. This represented 2% of foods for the Healthy Choices Framework and 3% of foods for the ADGs. All foods in the database could however be classified within the NOVA scheme.

**Table 1:**
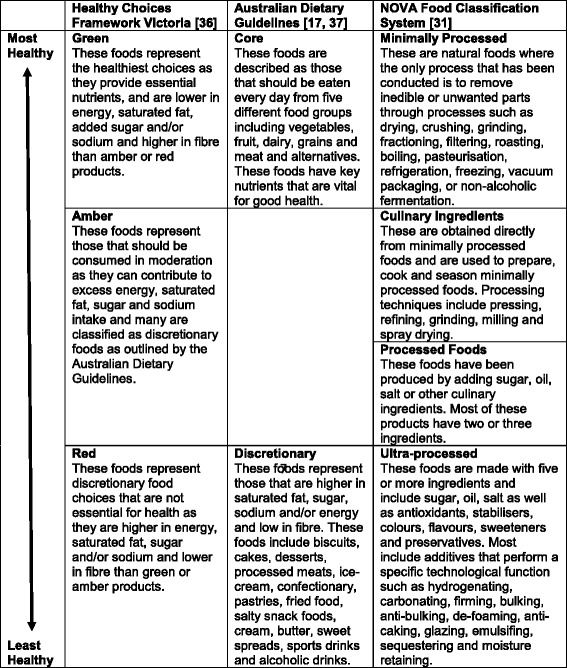
Classification of new food products launched in Australia in 2015 using three food classification schemes

A review was not required by the Institutional Human Research Ethics committee as this research was deemed negligible risk in accordance with the National Statement on Ethical Conduct in Human Research [38].

The first author (SAS) was responsible for completing all product classifications. A second researcher (JLW) independently checked these classifications for accuracy. Disagreements were resolved through consensus discussion amongst the full research team.

Descriptive statistics examined the proportion of new products both overall, and according to HAC member status, falling within each of the GNPD’s 17 food categories, and within the three food classification schemes.

Chi-squared tests were used to compare the proportion of Red or Green, Core or Discretionary and Ultra-processed or Minimally processed in HAC member products vs non-HAC member products. Analyses were conducted in SPSS (Version 23, IBM, St Leonards NSW), with *p* < 0.05 considered a statistically significant finding.

## Results

**Fig. 1:**
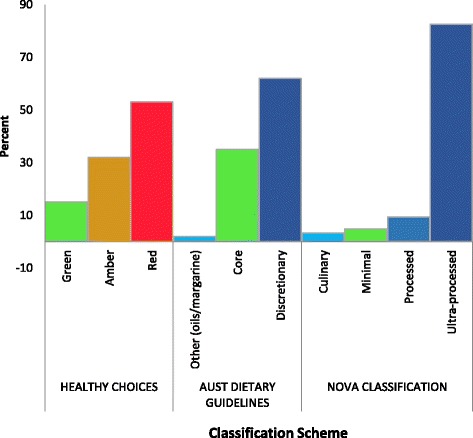
New food products launched in Australia in 2015, classified using three food classification schemes (*n* = 4143)^a^. ^a^Proportions may not add up to 100% as some foods such as condiments, baking products and oils were unable to be classified in the Healthy Choices Framework and Australian Dietary Guidelines

In 2015, 4143 new food products were launched into the Australian retail food market. Overall, 51% of new food products were classified as red, 62% as discretionary and 82% as ultra-processed (Fig. 1). In addition, a minority of foods were classified in each schemes’ most healthy category, i.e. green, core and minimally processed.

The breakdown of categories into which each food product belonged is shown in Table 2. The largest food category was bakery (16%), closely followed by snacks (15%). Sauces and seasonings comprised 10% of new products and dairy and chocolate confectionary each comprised 8%. These five categories represented over half (57%) of new food products released in 2015. Apart from the dairy category, the majority of new food products within each of these five categories were classified as red, discretionary and/or ultra-processed. Across all 17 categories, red foods and discretionary foods predominated in nine categories and ultra-processed foods predominated in all categories. Green foods predominated in only two categories (fruits and vegetables and soup), core foods were in the majority in eight categories and minimally processed foods only predominated in one category (fruits and vegetables). Fruits and vegetables represented just 3% of all new products released in 2015, and although over 80% of these were classified as green and all were core foods, 34% were classified as ultra-processed (Table 2).

**Table 2:** New food products launched in Australia in 2015 within each food category, classified using three food classification schemes (n = 4143)^a^

		Healthy Choices Framework Victoria *n* (%)			Australian Dietary Guidelines *n* (%)		NOVA Food Classification System *n* (%)			
Food Category	n (%)	Green	Amber	Red	Core	Discretionary	Minimal	Culinary	Processed	Ultra-processed
Bakery	676 (16)	78 (12)	135 (20)	454 (67)	108 (16)	559 (83)	26 (4)	6 (1)	28 (4)	616 (91)
Snacks	604 (15)	38 (6)	258 (43)	308 (51)	121 (20)	483 (80)	55 (9)	0 (0)	92 (15)	457 (76)
Sauces and Seasonings	421 (10)	28 (7)	197 (47)	109 (26)	44 (11)	255 (61)	21 (5)	58 (14)	47 (11)	295 (70)
Dairy	328 (8)	86 (26)	165 (50)	77 (24)	271 (83)	57 (17)	17 (5)	19 (6)	72 (22)	220 (67)
Chocolate Confectionary	324 (8)	0 (0)	0 (0)	324 (100)	0 (0)	324 (100)	0 (0)	0 (0)	0 (0)	324 (100)
Meals and Meal Centres	302 (7)	3 (1)	102 (34)	197 (65)	22 (73)	82 (27)	0 (0)	0 (0)	1 (1)	301 (99)
Processed Fish, Meat, Eggs	268 (7)	54 (20)	104 (39)	110 (41)	143 (53)	125 (47)	28 (10)	0 (0)	29 (11)	211 (79)
Deserts and Ice-creams	218 (5)	12 (6)	56 (26)	150 (69)	1 (1)	217 (99)	0 (0)	0 (0)	1 (1)	217 (99)
Sweets and Gum	215 (5)	0 (0)	15 (7)	200 (93)	0 (0)	215 (100)	0 (0)	0 (0)	0 (0)	215 (100)
Breakfast Cereals	164 (4)	53 (32)	84 (51)	27 (17)	150 (92)	14 (9)	10 (6)	0 (0)	21 (13)	133 (81)
Side Dishes	156 (4)	51 (33)	68 (44)	37 (24)	142 (91)	14 (9)	35 (22)	0 (0)	28 (18)	93 (60)
Fruit and Vegetables	107 (3)	86 (80)	21 (20)	0 (0)	107 (100)	0 (0)	39 (36)	0 (0)	34 (32)	34 (32)
Sweet Spreads	106 (3)	8 (8)	9 (9)	89 (84)	23 (22)	83 (78)	8 (8)	30 (28)	2 (2)	66 (62)
Soup	91 (2)	69 (76)	22 (24)	0 (0)	71 (78)	20 (22)	0 (0)	0 (0)	4 (4)	87 (96)
Savoury Spreads	79 (2)	27 (34)	36 (46)	16 (20)	0 (0)	70 (100)	0 (0)	0 (0)	0 (0)	70 (100)
Baby Food	58 (1)	23 (40)	32 (55)	3 (5)	52 (90)	6 (10)	8 (14)	0 (0)	25 (43)	25 (43)
Sweeteners and Sugar	26 (1)	0 (0)	5 (19)	21 (81)	0 (0)	26 (100)	0 (0)	17 (65)	0 (0)	9 (35)

^a^ Proportions may not add up to 100% as some foods such as condiments, baking products and oils were unable to be classified in the Healthy Choices Framework and Australian Dietary Guidelines

Seven percent (*n* = 297) of new food products were launched by HAC members (Campbell Arnott’s, Coca Cola, General Mills, Lion, Nestle, PepsiCo, Sugar Australia and Unilever) (Table 3). When compared to non-members, a significantly greater proportion of new food products launched by HAC members were classified as red (59% vs 51% for HAC members and non-members, respectively), discretionary (79% vs 61%), and ultra-processed (94% vs 81%) (all *p* < 0.001). In addition, of the new food products released by HAC members, a lower proportion were classified as green (8% vs 15%) for HAC members and non-members, respectively), core foods (18% vs 36%) and minimally processed (0% vs 6%) compared to the proportion launched by non-members (all *p* < 0.001) (Table 3).

**Table 3:** New food products launched in Australia in 2015 by HAC and non-HAC Members classified using three food classification schemes (n = 4143) ^a^

		Healthy Choices Framework Victoria *n* (%)			Australian Dietary Guidelines *n* (%)		NOVA Food Classification System *n* (%)			
Member	*n* (%)	Green	Amber	Red	Core	Discretionary	Minimal	Culinary	Processed	Ultra-processed
Non-HAC	3846 (93)	592 (15)*	1221 (32)	1948 (51)*	1401 (36)*	2325 (61)*	247 (6)*	121 (3)	376 (10)	3102 (81)*
HAC	297 (7)	24 (8)	88 (30)	174 (59)	52 (18)	234 (79)	0 (0)	9 (3)	8 (3)	280 (94)

*Abbreviations*: *HAC* Healthier Australia Commitment

^a^Proportions may not add up to 100% as some foods such as condiments, baking products and oils were unable to be classified in the Healthy Choices Framework and Australian Dietary Guidelines

^*^p < 0.001 - the proportion of items within Green, Red, Core, Discretionary, Minimally processed and Ultra-processed differed significantly for HAC and Non-HAC members as assessed by chi-square tests

## Discussion

Between January 1 and December 31 2015, 4143 new food products were launched into the Australian retail food market. According to nutrient-, food-, and processing-based criteria, the majority of these new products were classified in each schemes’ least healthy category (i.e. red, discretionary and ultra-processed). Products in these categories have been described as foods that should be either avoided or restricted in the diet because they are typically high in energy, saturated fat, sugar and/or sodium [17, 30, 36, 37], and are associated with increased risk of negative health outcomes such as obesity and chronic disease [4, 5]. In addition, a greater proportion of new products launched by companies that had publicly committed to improve the nutritional profile of their food product portfolios as part of the HAC were unhealthy, and a lower proportion were healthy, compared to products launched by companies that were not part of the HAC.

The current results are perhaps unsurprising given that most reformulation schemes are voluntary and not well taken up by the food industry [14, 20, 22, 39, 40]. The Australian Food and Health Dialogue is an example of a voluntary reformulation initiative that had minimal success in meeting its commitments to improve the nutrient profile of foods [41, 42]. This limited success was attributed to the voluntary nature of the scheme, which resulted in a lack of accountability when companies failed to reach their targets [41]. Similarly, the voluntary Responsibility Deal in the UK also yielded small but disappointing results in some respects, in that few pledges were met and limited positive impacts on dietary intake and health outcomes were observed [43, 44]. Whilst some reformulation programs have had some modest success, it should also be acknowledged that the strategy of producing “healthier” processed foods has limited ability to improve population level dietary intake [45, 46]. Although the current results do not show whether the nutritional quality of HAC members’ product portfolios changed (either positively or negatively) over the course of the commitment period, they nevertheless demonstrate that despite their nutrition-related commitments, the majority of new products released by HAC members were unhealthy in 2015. Greater monitoring of industry progress in improving the healthfulness of the food supply may therefore be warranted, with public accountability if the necessary changes are not seen.

Fruits and vegetables represented just 3% of the new products launched in 2015. Of these, the majority represented a healthier choice according to two schemes (i.e. 80% were classified as green and 100% were classified as core). However, according to the NOVA scheme just 36% were classified as minimally processed (predominantly frozen) and 32% were classified as ultra-processed. These findings suggest that manufacturers have little interest in launching new fruit and vegetable products, particularly minimally processed varieties. Limited availability may be contributing to low consumption rates, as only 4% of Australian adults consume the recommended daily serves of vegetables and 31% meet recommendations for fruit intake [47].

Whilst there have been no other known published studies of the healthfulness of new food products, it is worth comparing these results with others that have examined the healthfulness of the food supply. One study of packaged food products in supermarkets across New Zealand found that 83% of products were ultra-processed and had a poor nutrient profile [16]. Similarly, other studies have found a high percentage of unhealthy, highly processed foods available for purchase in large supermarkets in Australia, and this did not improve over time [48–50]. For instance, the availability of unhealthy processed products such as yoghurt and dairy desserts that were high in fat, sodium and/or sugar rose from 12% in 2005 to 23% in 2008 [50]. Another study investigated the nutritional composition of breakfast cereals and found no improvement from 2004 to 2010, with the majority remaining high in sugar [49]. In relation to snack foods and beverages available in large metropolitan supermarkets in Australia in 2004 (snacks) and 2006 (beverages), only 9–22% of snacks and 14–27% of beverages were deemed nutritious [48]. Studies have similarly shown that ultra-processed foods dominate the food supply in other nations, with over half of calories consumed in Canada and the US supplied by ultra-processed foods [14, 51, 52]. These results are concerning given that previous research indicates that food product availability influences food choice [6–8].

A role for consumers in driving food product development must also be acknowledged however, as the drivers of product development are multifactorial [19, 53]. While technological advancements and high profit margins have led the food industry to saturate the market with energy-dense, nutrient-poor, ultra-processed foods [14–16, 19, 25, 40, 53, 54], consumer demand for such foods has also increased due to factors such as economic growth, time constraints, and the influence of mass-marketing campaigns [19]. At the same time however, consumers are expressing a desire to eat more healthfully. According to a nationally representative survey Australians’ top dietary priorities in 2016 were to eat more fresh fruit and vegetables (40%), consume smaller portion sizes (31%), reduce sugar intake from food (24%), eat healthier snacks (23%) and reduce fat intake (23%) [55]. It is suggested therefore, that if the food industry want to be ‘part of the solution’, they have an important role in making tasty and affordable healthy food choices more widely available.

This study used a commercial database of new products to provide a comprehensive list of new foods introduced into the Australian retail food market in 2015. Although it is possible that some products were missed, it is unlikely to be a large number given the standardised and rigorous nature in which Mintel’s data are collected. It is important to note, however, that the GNPD only collects information on products positioned as “new” by companies in their communication to consumers, and companies who might reformulate do not always communicate this information to consumers in order to limit their rejection of new items (i.e. consumers may fear that the reformulated products may be inferior in taste). Although we may have missed identifying some reformulated products as a result, it is unlikely that missing these items would have impacted our results because reformulation typically involves very small changes. It is however possible, but unlikely, that such small changes could cause foods to change categories within the TLL scheme. In addition, reformulation would not move an item from being ultra-processed to processed, or from discretionary to core.

There are also several limitations of our analyses. It is possible that some product misclassifications occurred, however, having a second, independent researcher check all classifications and a consensus approach when there were differences should have limited any misclassifications. There were small percentages of foods for which no classification applied in the Healthy Choices Framework and the ADGs, however this would not influence our findings since these items cannot be deemed truly healthy or unhealthy. The fibre content was missing for the classification of 2.8% of products (mostly biscuits and classified red). This required seeking an alternative source (company website, AUSNUT 2011–13 food composition database and Calorie King) for this information. Any small errors this may have introduced are unlikely to have influenced findings.

The time period covered included the final year of the HAC, and it is possible that results may have differed had we analysed new products launched in other years. However, as 2015 was the last year of the commitment, it might be expected that HAC members would have launched significantly more new healthier products that year, given that they had 7 prior years in which to develop them. In addition, we did not assess changes pre- and post-HAC, and therefore although those making a commitment produced a higher proportion of unhealthy products compared to those not making a commitment, they may nevertheless have improved the nutritional quality of their product portfolios over the duration of the commitment period. The intent of this analysis was not to assess the success or failure of the HAC commitment, rather reference to the HAC was included to group companies on the basis of whether they had publicly indicated an intention to produce healthier food products, to determine if those who had made a healthy food commitment were in fact producing healthier food products than those who had not made such a commitment. Although two of the three classification schemes we used did not align with the specific HAC commitments, it is nevertheless instructive that those making a commitment failed to meet standards for “healthy” according to three schemes that represent how the public health community defines “healthfulness”, whereas those who made no commitment at all were more likely to meet standards for “healthy” according to these three schemes.

The statistical analysis included multiple chi-square tests comparing HAC and non-HAC members, and no adjustments were made for multiple testing. Adjustment would likely make little difference, however, cautious interpretation of the results is advised. Despite this, the results across three different classification schemes were highly consistent in showing that the majority of new products released into the Australian retail food market in 2015 were unhealthy according to three classification schemes.

## Conclusion

This study found that the majority of new products released into the Australian retail food market in 2015 were classified in each of three schemes’ least healthy categories. A greater proportion of new products launched by companies that publicly committed to improve the nutritional quality of their products and promote healthier food options were unhealthy, and a lower proportion were healthy, compared with new products launched by companies that did not so commit. Greater monitoring of industry progress in improving the healthfulness of the food supply may be warranted, with public accountability if the necessary changes are not seen.

## Abbreviations

ADG: Australian Dietary Guidelines
AFGC: Australian Food and Grocery Council
GNPD: Global New Products Database
HAC: Healthier Australia Commitment
